# Anti-Hu Antibody-Associated Adie's Pupil and Paraneoplastic Sensorimotor Polyneuropathy Caused by Primary Mediastinal Small Cell Carcinoma

**DOI:** 10.3389/fneur.2019.01236

**Published:** 2019-11-26

**Authors:** Lei Zhang, Si Luo, Hui Jin, Xueman Lv, Jiajun Chen

**Affiliations:** ^1^Department of Neurology, China-Japan Union Hospital of Jilin University, Changchun, China; ^2^Department of Ophthalmology, China-Japan Union Hospital of Jilin University, Changchun, China

**Keywords:** paraneoplastic neurological syndrome, anti-Hu antibody, small cell carcinoma, peripheral neuropathy, Adie's pupil

## Abstract

We report a woman with unilateral Adie's pupil associated with peripheral sensorimotor polyneuropathy, elevated anti-Hu antibody, and primary mediastinal small cell carcinoma (SCC). To our knowledge, this is the first report of Adie's pupil in a patient with mediastinal SCC. Although rare, Adie's pupil and sensorimotor polyneuropathy may be the first manifestation of cancer. Its rapid recognition facilitates an early diagnosis and treatment.

## Introduction

Adie's pupil is a neuro-ophthalmological disorder characterized by unilateral or bilateral tonically dilated pupils that do not respond to light but respond to accommodation (1). Its mechanism is not fully understood till now. Histological studies showed loss of ganglion cells in the parasympathetic ciliary ganglia, which resulted in supersensitivity of the iris sphincter muscle (2). Mostly, Adie's pupil runs a benign clinical course, but occasionally is related with paraneoplastic syndrome (3). Here, we report a woman complaining of the weakness of lower extremities. Further examinations showed unilateral Adie's pupil, sensorimotor polyneuropathy, elevated anti-Hu antibody, and primary mediastinal small cell carcinoma (SCC). After chemotherapy, with the resolution of the mediastinal mass, the weakness of lower extremities was improved a little, but the right tonic pupil continued. To our knowledge, this is the first report of Adie's pupil in a patient with mediastinal SCC.

## Case Presentation

A 50-year-old woman presented with a 2-year history of progressive weakness of lower extremities and her walking was subsequently impaired. She was a heavy smoker (20 cigarettes per day for 30 years). She had no symptoms of paresthesia or autonomic dysfunction and denied fever, fatigue, cough, dry mouth or eyes, rhinitis, anhidrosis, or weight loss. She did not complain of difficulties with her vision.

**Figure 1:**
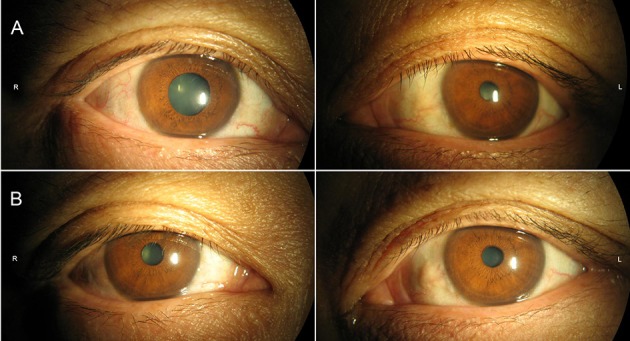
Pupils were mid-position in ambient light **(A)** without constriction in response to bright light. **(B)** Thirty minutes following diluted (0.0625%) pilocarpine solution, pupillary constriction of right eye was noted.

Neurological examination revealed a right-sided tonic pupil, 5 mm in diameter (Figure 1A) with no response to light. The left pupil measured about 3 mm and responded normally to light. At slit lamp, segments of the sphincter constrict (vermiform movements) could be observed. Thirty minutes after local instillation of one drop of diluted pilocarpine (0.0625%) in each eye, the right pupil constricted, whereas the left pupil remained unchanged (Figure 1B), a finding consistent with unilateral Adie's pupil. The strength of distal flexors and extensors of lower limbs was reduced (4/5, MRC scale), while the muscle strength of upper limbs and lower proximal limbs was normal. Sensory examination and position sense of the toes and fingers were normal. Generalized tendon areflexia existed, which could not be elicited with the Jendrassik maneuver. Plantar reflexes were flexor. Babinski's reflex was negative bilaterally.

**Figure 2:**
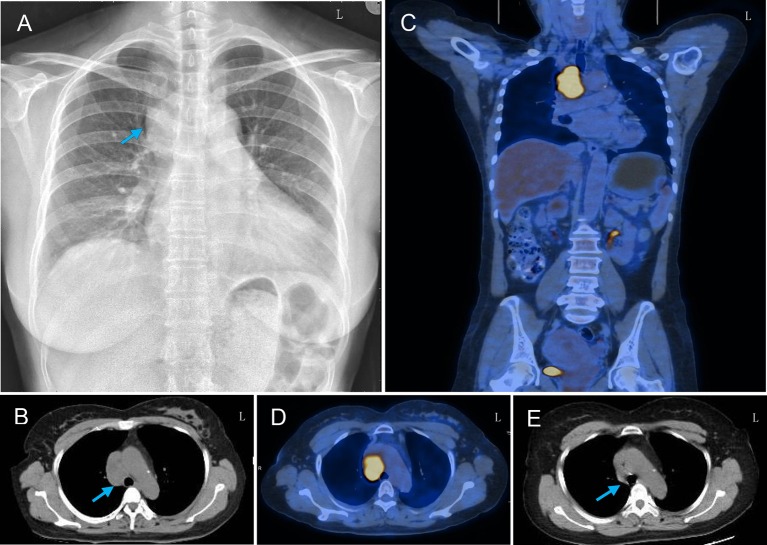
Thoracic radiograph and PET-CT findings of the patient. **(A)** Chest X-ray showed a round soft tissue mass (arrow) in the upper-mid mediastinum and infiltrating right lung field. **(B)** Thoracic CT showed a round soft tissue mass (arrow) located in the upper-mid mediastinum before the arteroae aorta. **(C,D)** PET-CT scan showed increased uptake of fluorodeoxyglucose signal in this mass axial and coronal plane images. **(E)** Repeated thorax CT showed the resolution of the mediastinal mass after chemotherapy.

A chest radiograph showed a round soft tissue mass located in the upper-mid mediastinum (Figure 2A). Motor and sensory nerve conduction studies of all limbs were conducted, and the results are shown in Table 1. The examination revealed an almost symmetrical motor neuropathy in the lower extremities (obviously prolonged latencies and low amplitudes of potentials). The latencies of motor median nerves were prolonged but their amplitudes were normal. These indicated an axonal motor polyneuropathy with possible demyelinating features based on the prolonged distal motor latencies. The asymmetrical reduction in amplitudes of sensory nerves in the four limbs could be observed. The conduction velocities of all the nerves tested were normal or minimally reduced (<10% below lower limit of normal). The F-wave parameters were all within the normal range and no conduction block was observed in the motor nerves tested. At the same time, needle electromyography did not reveal any abnormal findings. All these showed a sensorimotor polyneuropathy with lower extremities predominance.

**Table 1:** Nerve conduction study results of the patient.

	**Nerve**	**Latency (ms)**	**Normal limit**	**Amplitude (μV/mV)**	**Normal limit**	**NCV (m/s)**	**Normal limit**
Sensory^*^	Median R	1.89		28	>20	55	>50
	Median L	1.72		25	>20	56.3	>50
	Ulnar R	1.83		0.94↓	>20	51.9	>50
	Ulnar L	1.69		6.6↓	>20	56.2	>50
	Sural R	1.31		6.6↓	>18	42↓	>45
	Sural L	1.35		3.0↓	>18	48.1	>45
Motor^#^	Median R	8.06↑	<4	6.7	>4	51.3	>50
	Median L	7.5↑	<4	5.2	>4	51	>50
	Peroneal R	10.1↑	<6	0.21↓	>4	41.6↓	>45
	Peroneal L	12.0↑	<6	0.21↓	>4	41.9↓	>45

*NCV, nerve conductive velocity*.

* *Sensory nerve action potentials were antidromically obtained*.

# *Compound motor action potentials of the median nerves and peroneal nerves were recorded from the abductor policis brevis and extensor digitorum brevis with the stimulating electrodes on wrist and ankle, respectively. Their motor conductive velocities were measured between the wrist and elbow and between the ankle and knee, respectively*.

*↑:Higher than normal limit*.

*↓:Lower than normal limit*.

**Figure 3:**
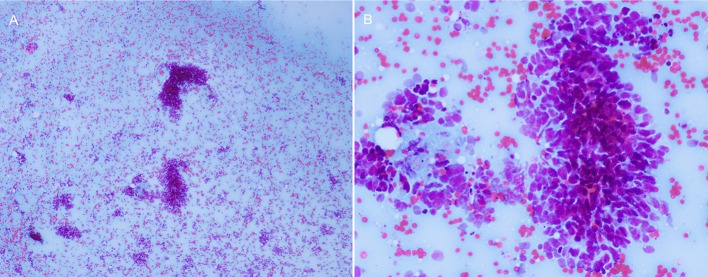
The cytology of the mediastinal mass showed small round/oval cells with high nuclear/cytoplasmic ratio, consistent with primary SCC. **(A)** 40×, **(B)** 100×.

To evaluate the possibility of a paraneoplastic neurological syndrome (PNS), serological tests for paraneoplastic antibodies were carried out and showed a positive titer for anti-Hu antibody. A thoracic computed tomography (CT) scan showed a round soft tissue mass (3.6 × 2.7 × 4.5 cm) located in the upper-mid mediastinum (Figure 2B). Whole-body positron emission tomography (PET)-CT demonstrated a significant increase in the fluorodeoxyglucose signal in this round mass (Figures 2C,D). With the help of chest physicians, an endobronchial ultrasound bronchoscopy aspiration of the mass confirmed the presence of primary SCC by cytology (Figures 3A,B). A diagnosis of Adie's pupil and paraneoplastic sensorimotor polyneuropathy associated with anti-Hu antibodies was made.

Because of its special location, the carcinoma could not be resected by surgery. The patient was referred to the oncology department to initiate first-line chemotherapy with cisplatin and etoposide. At the same time, she was given intravenous methylprednisolone (20 mg/day). Three months after the diagnosis and receiving chemotherapy for three cycles, repeated thorax CT showed the resolution of the mediastinal mass (Figure 2E). The weakness of lower extremities was improved a little, but the right tonic pupil and the tendon areflexia continued.

## Discussion

Tonic pupils is usually a benign condition, occurring more frequently in women (Adie's pupil), and may be associated with depressed or absent muscle stretch reflexes (Holmes-Adie syndrome), impaired sweating (Ross syndrome), and isolated hemifacial anhidrosis (Harlequin syndrome) (4). However, rare cases may be secondary to the remote effects of malignancy typical in conjunction with sensory neuropathy. It mostly occurs in the context of small-cell lung carcinoma associated with type 1 antineuronal nuclear autoantibodies (anti-Hu) (3, 5–10).

PNSs are mostly a consequence of cross-reactivity between tumor and host antigens. Subacute sensory neuropathy is the most frequent presentation of PNS in patients with anti-Hu antibodies (11–13), and the typical electrophysiological pattern is marked sensory nerve conduction reduction with normal motor nerve conduction. Biopsy of the sural nerve revealed demyelination and peripheral nerve microvasculitis (14). However, some other studies showed a different pattern (12, 15, 16). In 16 patients with positive anti-Hu antibodies, the most common clinical feature was sensorimotor neuropathy, accounting for 50% of cases. Sural nerve biopsy showed axonal degeneration as the most characteristic feature and inflammatory cells in 43% of cases (17). In Camdessanche's series, axonal/demyelinating pattern was more frequent in motor nerves and axonal/neuronal pattern in sensory nerves (11). The current patient presented with the weakness of lower extremities and unilateral Adie's pupil; the nerve conduction study revealed an axonal sensorimotor neuropathy with possible demyelinating features. These presentations were not typical at all, indicating that neuropathy associated anti-Hu antibody can be heterogeneous and waits more clinical and pathological evidences.

Furthermore, in most of the patients, recognition of PNS is difficult since its manifestations are sometimes the first and the only sign of disease, just as in this case. Usually, these symptoms are not specific and occur long before the diagnosis of carcinoma (18). So, in patients with Adie's pupil and sensorimotor polyneuropathy, a PNS should be considered as a differential diagnosis. Extensive screening of onconeural antibodies is important to establish/confirm the diagnosis and to evaluate its prognosis.

SCC, a kind of neuroendocrine carcinoma, is found in about one in four patients with lung cancer (19), although it can also occur in multiple extrapulmonary organs including gastrointestinal tract, thymus, parathyroid gland, ovaries, and biliary system. However, mediastinal primary SCC is extremely rare. Its origin is not clear (20). The tumor cells could synthesize, store, and release peptide or amine hormones such as gastrin and chromogranin. These hormones can cause specific symptoms, which are typically referred to as carcinoid syndrome and include flushing, abdominal pain, diarrhea, asthma, and heart symptoms. Of note, the diagnosis of SCC is primarily based on H&E morphology and does not require demonstration of neuroendocrine markers by immunohistochemistry (21).

## Conclusion

The present case highlights the importance of considering rare associations as possible diagnoses. Of particular importance is the fact that Adie's pupil and sensorimotor polyneuropathy might be the first sign of carcinoma and deserve a comprehensive neurological and electrophysiological examination.

## Data Availability Statement

The datasets generated for this study are available on request to the corresponding author.

## Ethics Statement

This study has been reviewed and approved by the Ethics Committee of the China–Japan Union Hospital of Jilin University. The patient had provided written informed consent to the publication of this case, including the photos in accordance with the Declaration of Helsinki.

## Author Contributions

LZ was responsible for drafting the manuscript. SL and HJ were responsible for conducting serological testing and interpreting the results. XL was responsible for the ocular examination. JC was responsible for study concept or design, drafting/revising the manuscript, and the final approval.

### Conflict of Interest

The authors declare that the research was conducted in the absence of any commercial or financial relationships that could be construed as a potential conflict of interest.
